# Supplementary material for: Assessing the mechanism and therapeutic potential of modulators of the human Mediator complex-associated protein kinases
Source: eLife. 2016 Dec 9;5:e20722. doi: 10.7554/eLife.20722 (PMC5224920; doi:10.7554/eLife.20722)
Supplement: Figure 1—source data 1. — DOI: http://dx.doi.org/10.7554/eLife.20722.004 [file elife-20722-fig1-data1.docx]

|  | 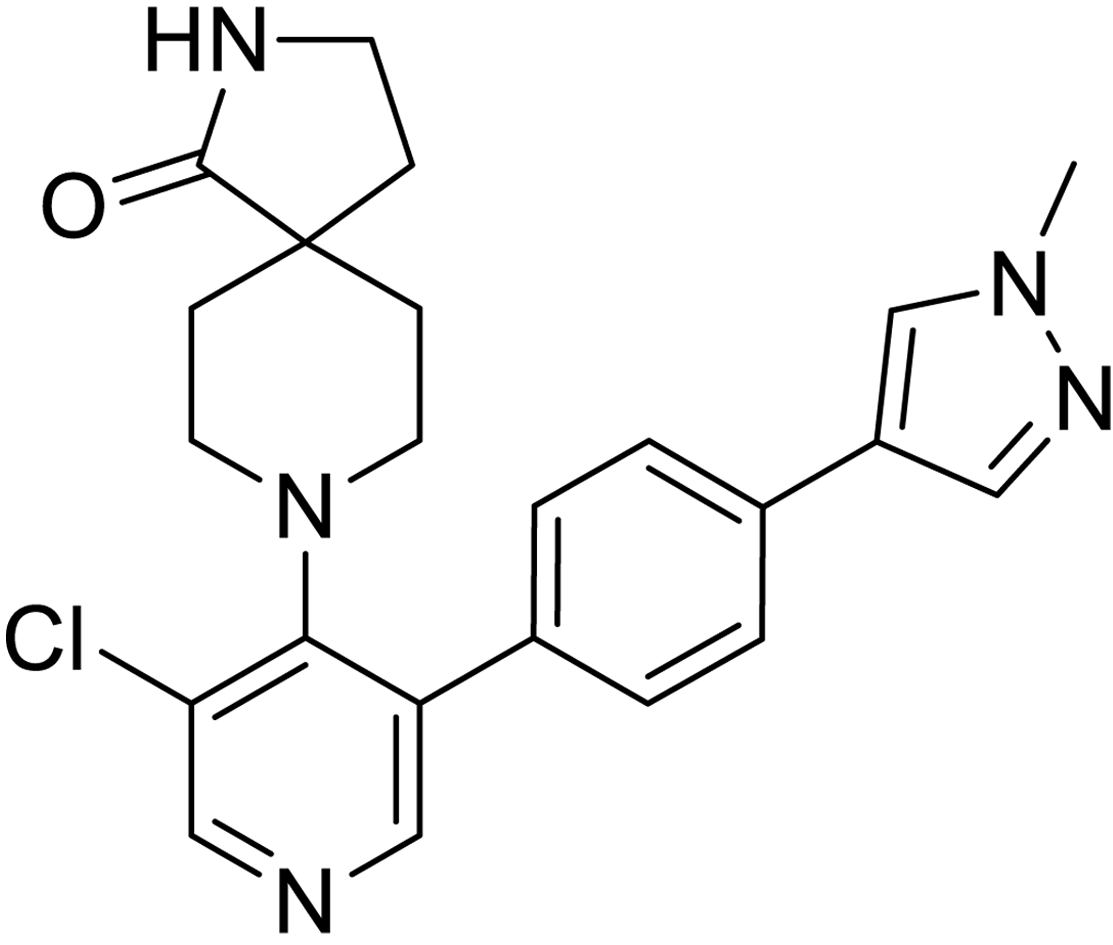 | 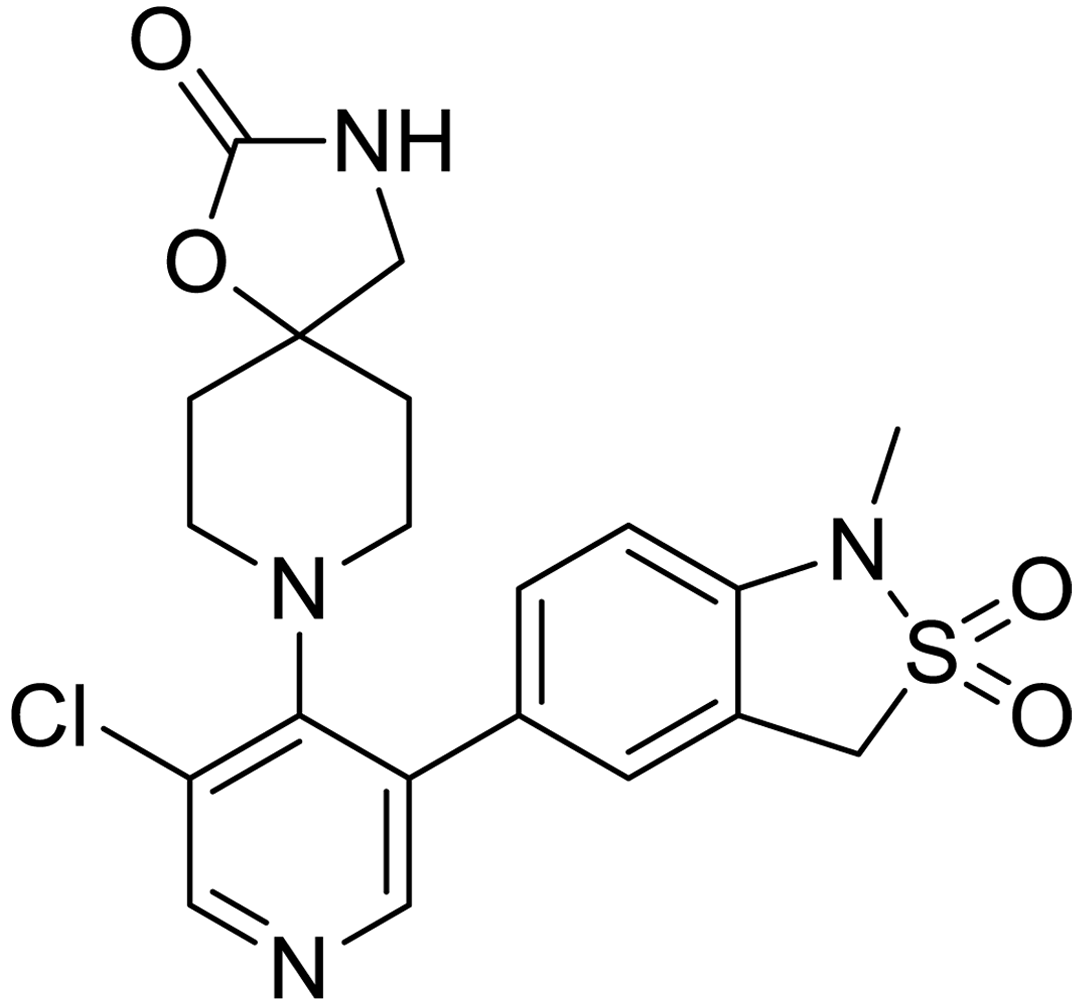 | 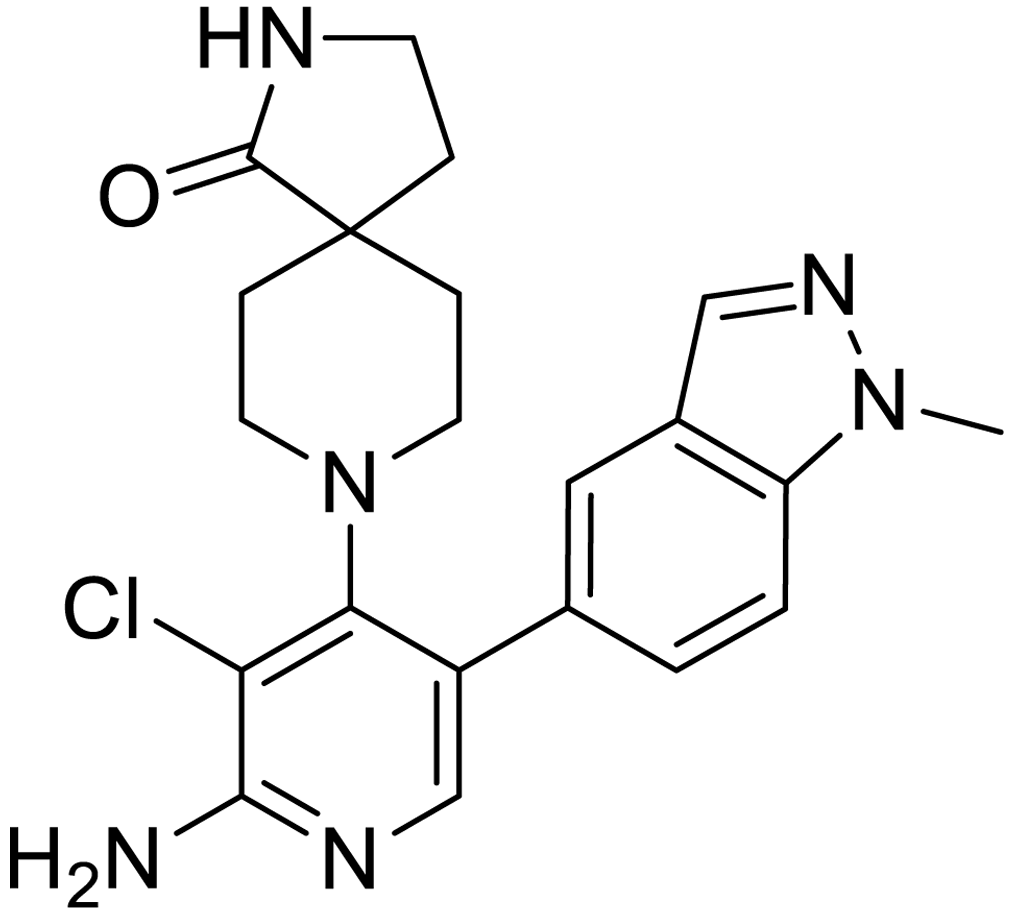 | 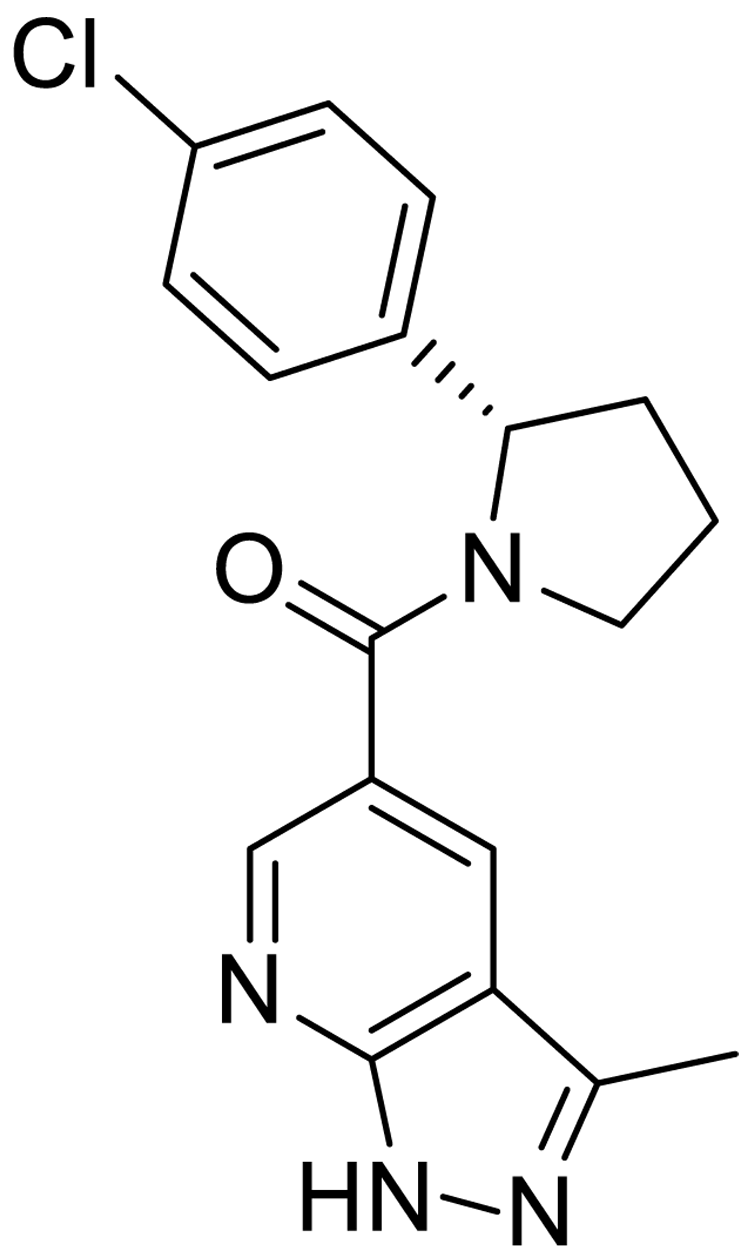 | 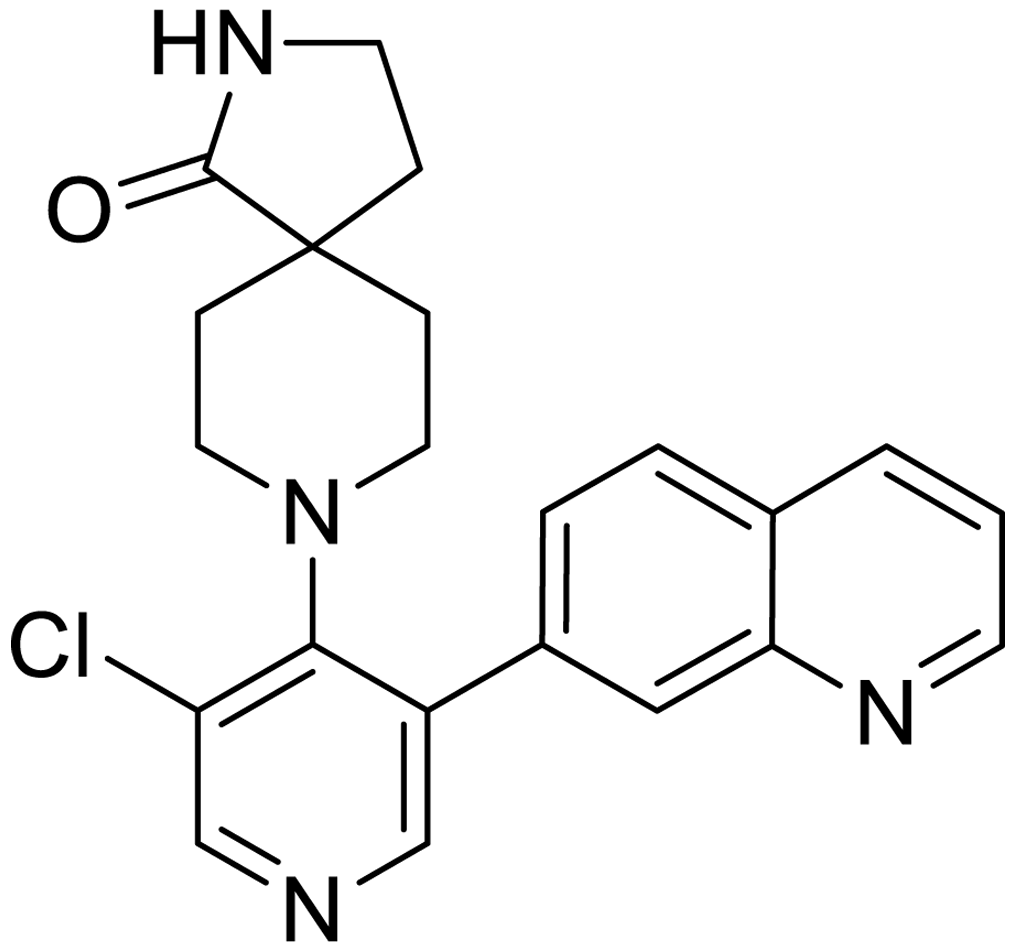 | 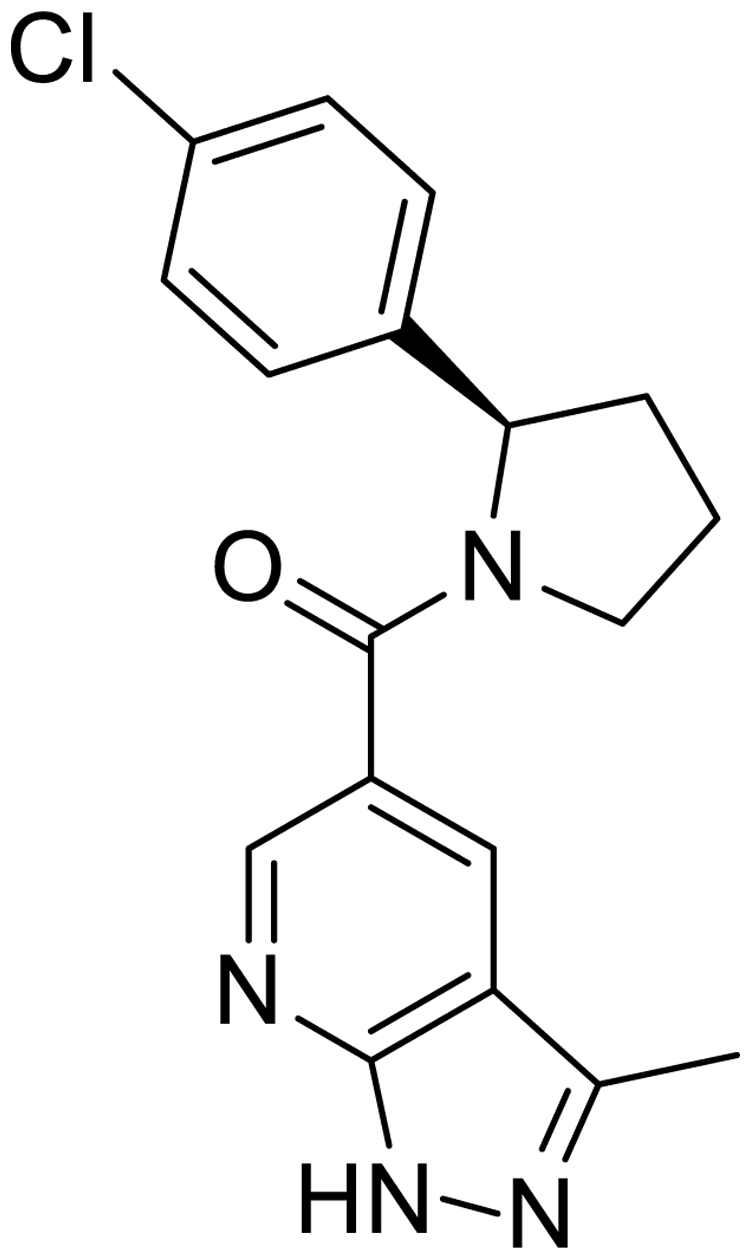 |
| --- | --- | --- | --- | --- | --- | --- |
|  | **CMPD 1** | **CMPD 2** | **CMPD3** | **CMPD 4** | **CMPD 5** | **CMPD 6** |
| CDK8 IC_50_(nM) | 5.0 ± 1.0 | 1.8 ± 0.2 | 4.9 ± 0.6 | 4.4 ± 0.3 | 103 ± 19 | 590 |
| CDK19 IC_50_(nM) | 6.0 ± 1.0 | 1.6 ± 0.2 | 2.6 ± 0.4 | 3.8 ± 0.4 | 72 ± 10 | nd |
| p-STAT1^SER727^ IC_50_ (nM) | 23.3 ± 14.8 | 4.7 ± 5.5 | 25.5 ± 12.0* | 7.7 ± 2.2 | 330 ± 127* | nd |
| 7dF3 IC_50_ (nM) | 5.0 ± 2.0 | 1.4 ± 0.8 | 11.8 ± 17.9 | 5.3 ± 2.0 | 1430 ± 760 | 2100 |
| LS174T IC_50_ (nM) | 23.1 ± 10.9 | 10.8 ± 4.2* | 33.3 ± 12.8 | 36.2 ± 9.5* | nd | nd |
| **Caco-2** |  |  |  |  |  |  |
| Papp A-B (cms^-1^) | 23.7 | 16.6 | 31.8 | 43.5 | nd | nd |
| Efflux ratio | 2.5 | 4.7 | 2.4 | 1.49 | nd | nd |
| **Mouse PK** |  |  |  |  |  |  |
| Cl (L/h/kg) | 1.87 | 2.86 | 0.61 | 1.37 | nd | nd |
| Vd (L/kg) | 1.08 | 1.34 | 0.63 | 0.59 | nd | nd |
| F (%) | 54 | 17 | 30 | 25 | nd | nd |
| **Rat PK** |  |  |  |  |  |  |
| Cl (L/h/kg) | 1.54 | 3.00 | 1.49 | 1.50 | nd | nd |
| Vd (L/kg) | 1.53 | 2.82 | 1.99 | 1.62 | nd | nd |
| F (%) | 88 | 12 | 57 | 66 | nd | nd |
| **Dog PK** |  |  |  |  |  |  |
| Cl (L/h/kg) | 0.84 | 1.93 | 1.07 | 1.35 | nd | nd |
| Vd (L/kg) | 0.74 | 1.29 | 1.4 | 1.47 | nd | nd |
| F (%) | 126 | 43 | 68 | 39 | nd | nd |
| **Kinase selectivity** |  |  |  |  |  |  |
| No. of kinases | 3/293 | 1/293 | 0/279 | 1/264 | nd | nd |

**Table 1. Properties of CDK8/19 ligands.** Mean values from at least three (± s.d.) or *two (± range) independent repeats (nd = not determined). The number of protein kinases inhibited by > 50% in response to 1µM test compound is indicated in the last row. IC_50_ values were determined for off-target activity where >50% inhibition against a protein kinase was detected. These were GSK3α (462 nM), GSK3β (690 nM) and PRKCQ (122 nM) for **1**, TRK3B (82 nM) for **2** and GSK3α (691 nM) for **4**, as compared to single digit nM IC_50_ values for all active compounds tested against CDK8 and CDK19. No off-target activities were detected for **3**. Mouse, rat and dog PK experiments were run with 0.2mg/kg i.v. or 0.5mg/kg p.o. for each of the active compounds.

|  |  |  |  |  |
| --- | --- | --- | --- | --- |
|  | **SEL120-1 (Selvita)^a^** | **Senexin A**  **(Senex)^b^** | **Senexin B**  **(Senex)^c^** | **Compound 48**  **(Roche)^d^** |
| CDK8 IC_50_ (nM) | 14.6 ± 0.9 | 86.0 ± 5.3 | 28.9 ± 3.3 | 1.8 ± 0.1 |
| CDK19 IC_50_ (nM) | 8.1 ± 0.7 | 111.0 ± 15.2 | 30.4 ± 1.7 | 3.1 ± 0.1 |
| p-STAT1^SER727^ IC_50_ (nM) | 140 ± 28* | 730 ± 113* | 740 ± 14* | 1.3 ± 1.0* |
| 7dF3 IC_50_ (nM) | 47.0 ± 14.8* | 340 ± 127* | 160 ± 49* | 0.2 ± 0.02* |

**Table 2. IC_50_ values of published CDK8 type I ligands.** Mean values from at least three (± s.d.) or *two (± range) independent repeats (nd = not determined). Source literature – a: WO2014072435; b: US2012007147, US20090281129; c: WO2013116786; d: WO2014090692, WO2014029726.

|  |  | **COLO205** | **SW948** | **HT29** | **LS513** | **DLD1** | **LS180** | **LS174T** | **RKO** | **SW620** | **SW837** |
| --- | --- | --- | --- | --- | --- | --- | --- | --- | --- | --- | --- |
| **Wnt pathway**  **mutations** | **APC/**  **CTNNB1** | APC T1556fs*3 | APC Q1429*x  R1114* | APC  T1556fs*3  .E853* | CTNNB1  A5_  A80del | APC  P1427* | APC  R1788C  CTNNB1  S45F | CTNNB1  S45F | WT | APC  Q1338* | APC  R1450*  R213* |
| **Gene copy no. (log_2_)** | ***CDK8*** | 2.13 (2.28) | 2.13 (1.89) | 1.78 (2.14) | 1.73 (nd) | 1.00 (0.93) | 0.99 (nd) | nd (0.94) | 0.94 (0.93) | 0.65 (0.67) | 0.55 (0.51) |
|  | ***CDK19*** | 0.64 (0.63) | 0.94 (1.01) | 0.89 (0.67) | 0.90 (nd) | 1.01 (0.98) | 1.00 (nd) | nd (0.98) | 0.95 (0.93) | 1.02 (1.03) | 0.54 (0.53) |
| **Relative protein exp^n^** | **CDK8** | 106 (2) | 241 (68) | 38 (7) | 27 (29) | 17 (3) | 50 (13) | 62 (15) | 74 (11) | 48 (12) | 116 (7) |
|  | **CDK19** | 83 (1) | 2394 (41) | 93 (16) | 109 (41) | 67 (1) | 365 (41) | 127 (21) | 481 (91) | 210 (12) | 1242 (17) |
| **24 hour TCF/LEF Reporter assay IC_50_ (nM)** | **1** | 19 (2) | nd | nd | nd | 54 (13) | 25 (3) | 34 (5) | > | nd | 17 (2) |
|  | **2** | 8 (1) | nd | nd | nd | 63 (11) | 8 (1) | 20 (2) | > | nd | 6 (1) |
|  | **3** | 15 (3) | nd | nd | nd | 173 (28) | 28 (3) | 39 (6) | > | nd | 14 (1) |
|  | **4** | 9 (1) | nd | nd | nd | 97 (9) | 31 (5) | 31 (2) | > | nd | 8 (1) |
|  | **5** | 694 (148) | nd | nd | nd | 6134 (239) | 2127 (579) | 2561 (280) | > | nd | 777 (152) |
|  | **6** | > | nd | nd | nd | > | > | > | > | nd | > |
| **96 hour GI_50_ (nM)** | **1** | > | > | > | > | > | > | > | > | > | 6797 (1537) |
|  | **2** | > | > | > | > | > | > | > | > | > | > |
|  | **3** | > | > | > | > | > | 6667 (3395) | > | > | 1857 (498) | 3109 (710) |
|  | **4** | > | > | > | > | > | > | > | > | > | > |
|  | **5** | > | > | > | > | > | > | > | > | > | > |
|  | **6** | > | > | > | > | > | > | > | > | > | > |
| **14 d colony growth (350 nM; relative to control)** | **1** | nd | 0.40 (0.02) | 0.49 (0.04) | 0.05 (0.01) | 0.67 (0.01) | 0.18 (0.30) | 0.36 (0.03) | 1.09 (0.06) | 0.29 (0.11) | 0.72 (0.10) |
|  | **2** | nd | nd | nd | nd | nd | nd | nd | nd | nd | nd |
|  | **3** | nd | 0.45 (0.01) | 0.50 (0.07) | 0.02 (0.01) | 0.70 (0.03) | 0.13 (0.01) | 0.30 (0.03) | 0.95 (0.08) | 0.27 (0.06) | 0.79 (0.07) |
|  | **4** | nd | 0.13 (0.02) | 0.43 (0.01) | 0.04 (0.01) | 0.66 (0.07) | 0.13 (0.03) | 0.36 (0.01) | 0.98 (0.07) | 0.17 (0.01) | 0.54 (0.03) |
|  | **5** | nd | 1.02 (0.01) | 1.06 (0.14) | 1.22 (0.06) | 0.89 (0.03) | 1.13 (0.63) | 0.93 (0.02) | 0.94 (0.04) | 0.82 (0.04) | 0.95 (0.03) |
|  | **6** | nd | 0.74 (0.03) | 1.13 (0.17) | 1.23 (0.05) | 0.98 (0.01) | 0.74 (0.11) | 0.93 (0.11) | 0.96 (0.10) | 0.96 (0.10) | 0.95 (0.09) |

**Table 3. Effect of compounds on reporter expression and cell proliferation in a human colorectal cancer cell line panel.** Gene copy numbers for *CDK8* and *CDK19* were taken from the Cancer Cell Line Encyclopedia dataset (<http://www.broadinstitute.org/ccle/data>) or generated by array CGH (data in brackets). CDK8 and CDK19 protein levels were determined by bead-based ELISA. IC_50_ and GI_50_ are mean values ± s.d., n = 4. SW948, LS513 and SW620 had insufficient basal reporter assay activity for determination of IC_50_. Colony growth values are mean ± range, n = 2. > = value greater than 30µM, nd = not determined. COLO205 have poor adherence properties that prevented their use in clonogenic assays.
